# Tocopherol Content and Fatty acid Profile of Different Iranian Date Seed Oils

**Published:** 2012

**Authors:** Mahmood Biglar, Mahnaz Khanavi, Mannan Hajimahmoodi, Shokufeh Hassani, Ghazaleh Moghaddam, Naficeh Sadeghi, Mohammad Reza Oveisi

**Affiliations:** a *Pharmaceutical Sciences Research Center, Tehran University of Medical Sciences, Tehran, Iran.*; b *Department of Pharmacognosy and Traditional Iranian Pharmacy Research Center, Faculty of Pharmacy, Tehran University of Medical Sciences, Tehran, Iran.*; c *Department of Drug and Food Control, Faculty of Pharmacy, Tehran University of Medical Sciences, Tehran, Iran.*

**Keywords:** Date, Fatty acid, Iranian, *α*-tocopherol, GC, HPLC

## Abstract

Date is one of the world’s oldest food-producing plants wich has always played an important role in the economy and social life. Various researchers examined chemical composition and nutritional values of edible parts of dates while limited information about chemical composition and nutritional quality of date seed is available. In this study, fatty acid composition and total tocopherol content of 14 Iranian date seed oils were studied. Statistical analysis was performed through SPSS computing package. According to the fatty acid profiles, seven fatty acids were found through nearly 50% oleic acid in seeds. Shekar cultivar by 51.40% had the maximum amount and Lasht cultivar by 33.38% had the minimum amount of oleic acid. Tocopherol content in the samples varied between 33.86 μg vit E/g oil for Shahabi2 to 10.09 μg vit E/g oil for Shekar. Tocopherol content was 1.88 and 0.61 μg respectively in one-gram seed of these two cultivars. Iranian date seed oils classified as oleic-lauric oil, had a high amount of oleic acid and could serve as a profitable source of valuable oils for industrial applications.

## Introduction

In Iran, date is produced in abundance with approximately 1 million tons of production, being among the largest date producers after the Egypt. Dates have originated around the Persian Gulf (1). Iran is one of the date producing countries that approximately 185000 hectares of its land is under date cultivation. Hormozgan province, especially Haji-Abad region is the main producing area in Iran (2).

Date seed oil can be used in cosmetics, pharmaceuticals and food products (3). Previous researches have shown that date’s palm seed can provide suitable milled without any undesirable effects on sensory bread quality as a fiber source (4, 5). Indeed, 10-15% of date’s palm weight is average weight of its seed (4). A large quantity of date seed could be easily collect from the date processing industries or from the waste products originating. Various researchers (4, 6-11) examined chemical composition and nutritional value of edible part of dates while limited information about nutritional quality of date seed oil is available. Today, this functional edible oil is one of the 17 major oils and fats produced in the world, which increases the health-enhancing nutrients by its antioxidants (12, 13).

It has been hypothesized that the date seed oils are rich in tocopherols and can be considered as one of the sources of vitamin E securing. Moreover, fatty acids are important factor, which have directly effect on quality of date oils (13).

Due to this importance, free fatty acid composition and tocopherols content of the 14 Iranian date seed oils were analyzed through gas chromatography and high performance liquid chromatography, respectively.

## Experimental

*Seed materials*

Fourteen different date cultivars consist of Shahabi2, Shahabi, Goftar, Zahedi, Kabkab, Shekar, Dalki 2, Majul, Khenizi, Sayer, Lasht, Shahani, Maktub and Khasuee obtained from a local farm in Bushehr, Iran, at the beginning of harvest season in 2006. The shape, size and color of each variety were completely different. Voucher specimens had identified and deposited in the Herbarium of the Agricultural Research and Natural Resource Center of Bushehr province, Bushehr, Iran. They were dispatched through the airplane to Tehran University of Medical Sciences. Mature fruits of uniform size, free of physical damage and injury from insects and fungal infection, were selected and used for all experiments. Upon arrival at the laboratory, the samples (500 g portions) were packed in polyethylene bags, sealed and stored at 2-8°C until analysis.

*Lipid extraction*

Twenty seeds of each date cultivars were washed with distilled water, dried and then grounded to a fine powder. The lipid of each seed cultivars (20 g) was extracted using *n*-hexane in a soxhlet apparatus for 8 h. The lipids were weighed after the solvent evaporation and then stored at -10°C until analysis.

*Tocopherol analysis*

For tocopherol analysis, 0.2 g of each extracted oils were weighed, 5 mL of ascorbic acid solution (0.1 M) and 20 mL of potassium hydroxide solution (2 M) were added and heated at 60°C for 45 min. After filtering the mixture, 10 mL of saturated sodium chloride solution and 10 mL of butanol in *n*-hexane (5 mg/L) were added and then, samples were stirred by vortex mixer for 1 min. The hexane phases including tocopherols were dried through adding anhydrous sodium sulfate.

The residue was re-extracted by 5 mL *n*-hexane. This new fraction was combined with the first extract and the hexane residue flushed by nitrogen stream. The samples were re-dissolved in 1 mL of methanol, filtered and kept at -20°C until analysis. The sample’s tocopherol content was analyzed using UV-HPLC system and Eurospher 100-C8 column (4.6 mm × 25 cm) eluted isocratically with the acetonitrile : water (93 : 7) with a flow rate of one mL/min. The wavelength was set up at 294 nm.

*Fatty acid composition*

For fatty acid analysis, 0.5 g of each oil cultivar was weighed and 5 mL of NaOH (50 mg/mL) in isopropanol was added and heated in boiling water bath for 30 min. After evaporating the isopropanol and saponifying the oils, 0.35 mL of H_2_SO_4_: H_2_O (2 : 1) was wisely added to the soap drop.

A volume of 10 mL hexane : butanol (100 : 1) solution containing octadecanoic acid ester (3 mg/mL) as an internal standard was added to the tube. The solution was filtered through a 0.45 μm filter and a volume of 1 μL was injected.

The fatty acids were analyzed using GC (Dani, 1000 DPC model) fitted with a capillary column AT-1000 (25 m × 0.32 mm id, 0.2 μm film thicknesses). The injector and detector temperatures were set at 250°C and 280°C, respectively. The initial oven temperature was held for 2 min at 180°C followed by ramping at 22°C/min to 240°C and then held again for 25 min (14).

## Results and Discussion

The total fat, the saturated and unsaturated fatty acid contents, the ratio between unsaturated and saturated fatty acids (USFA/SFA ratios) and the Cox value of each date seed oils are presented in Table 1. 

**Table 1:** Total fat, saturated and unsaturated content and oxidative stability of 14 different Iranian date seed oils

**Cultivar**	**Fat content (%)**	**SFA (%)**	**USFA (%)**	**USFA/SFA (%)**	**Cox value (%)**
Shahabi2	5.57	41.35±0.32^c^	49.19±0.56^c^	1.18±0.01^a^	1.07±0.02^b^
Sh Shahabi	4.83	51.04±0.40^b^	48.18±0.38^c^	0.94±0.01^b^	1.05±0.01^b^
Goftar	6.27	48.47±0.09^b^	51.52±0.09^b^	1.06±0.00^b^	1.24±0.02^c^
Zahedi	6.28	49.41±1.51^b^	50.58±1.51^b^	1.02±0.06^b^	1.09±0.02^b^
Kabkab	4.60	54.30±1.83^b^	45.69±1.83^c^	0.84±0.06^c^	1.14±0.09^b^
Shekar	6.05	42.74±1.20^c^	57.25±1.20^a^	1.34±0.06^a^	1.11±0.03^b^
Kabkab Dalki 2	6.01	50.93±0.37^b^	49.06±0.37^b^	0.96±0.14^b^	1.15±0.01^b^
Majul	7.01	47.54±0.99^b^	51.93±1.00^b^	1.09±0.04^b^	1.20±0.02^b^
Khenizi	5.92	48.40±1.09^b^	51.10±1.17^b^	1.05±0.04^b^	1.13±0.02^b^
Sayer	7.11	49.26±0.61^b^	50.73±0.61^b^	1.03±0.02^b^	1.14±0.01^b^
Lasht	6.71	52.81±4.11^b^	38.75±0.44^d^	0.73±0.05^c^	0.88±0.01^a^
Shahani	6.45	60.17±0.89^a^	39.82±0.89^d^	0.66±0.02^d^	0.90±0.01^a^
Maktub	7.72	51.22±0.84^b^	48.30±0.80^c^	0.94±0.03^b^	1.07±0.01^b^
Khasuee	4.62	49.99±1.43^b^	50.00±1.43^b^	1.00±0.05^b^	0.99±0.02^b^

Values in the same column bearing different superscripts are significantly (p ≤ 0.05) different. SFA: Saturated fatty acid, USFA: Unsaturated fatty acid

The highest percentage of oil was in Maktub cultivar whilst the minimum amount was found in Kabkab. It can be seen that Shekar cultivar was rich in USFA and Shahani had the maximum percentage of SFA due to its high level of lauric acid. The fatty acid profiles in 14 different Iranian date seed oil can be seen in Table 2. Within each type of fatty acid, there were small variations but at least six identifiable peaks with suitable baseline separations can be illustrated from gas chromatogram. The major founded fatty acids in the samples were lauric, myristic, palmitic, stearic, oleic and linoleic acid. Oleic acid made up nearly half of the fat in most of the seeds and ranged from 33.38% for Lasht cultivar to 51.40% for Shekar. This finding was in well agreement with those reported in the literature (3, 15, 16). The second prevalent fatty acid was lauric acid by 24.35% mean content, ranged from 18.78% for Shekar cultivar to 31.61% in Shahani.

**Table 2:** Fatty acid profile of 14 different Iranian date seed oils

**Cultivar**	**Luric %**	**Myristic %**	**Palmitic %**	**Palmitoleic %**	**Stearic %**	**Oleic %**	**Linoleic %**	**Other %**
Shahabi 2	23.14±0.13^b^	1 5.33±0.16^b^	10. 34±0.20^b^	0.0	2.52±0.08^a^	42. 87±0.16^b^	6.31±0.12^b^	0.0±0.00
Shahabi	23.33±0.19^b^	1 4.73±0.16^b^	10.79±0.11^b^	0.0	2.17±0.07^a^	41.98±0.24^b^	6.19±0.143^b^	0.77±0.07^a^
Goftar	23.22±0.07^b^	12.42±0.03^c^	10.26±0.17^b^	0.08±0.02^a^	2.55±0.08^a^	43.55±0.13^b^	7.88±0.21^a^	0.00±0.00
Zahedi	25.95±1.04^b^	11.42±0.59^c^	9.59±0.557^b^	0.000	2.44±0.41^a^	44.29±1.74^b^	6.29±0.33^b^	0.00±0.00
Kabkab	25.80±1.95^b^	12.46±0.82^c^	12.14±0.50^a^	0.87±1.28^a^	3.89±2.47^a^	37.28±1.60^c^	7.54±0.85^a^	0.00±0.00
Shekar	18.78±0.91^b^	10.22±0.22^d^	10.70±0.20^b^	0.000	3.03±0.29^a^	51.40±1.13^a^	5.85±0.26^c^	0.00±0.00
Kabkab Dalki 2	25.58±0.08^b^	11.68±0.07^c^	11.38±0.30^a^	0.000	2.29±0.11^a^	41.96±0.60^b^	7.09±0.24^a^	0.00±0.00
Majul	23.39±0.67^b^	11.26±0.30^c^	9.78±0.17^c^	0.000	3.09±0.12^a^	44.53±0.85^b^	7.40±0.15^a^	0.51±0.16^a^
Khenizi	24.04±0.92^b^	11.69±0.21^c^	10.27±0.10^b^	0.000	2.38±0.04^a^	44.38±1.03^b^	6.71±0.16^b^	0.48±0.10 ^a^
Sayer	23.59±0.81^b^	13.04±0.19^c^	10.79±0.21^b^	0.000	1.84±0.25^a^	43.88±0.50^b^	6.85±0.14^b^	0.00±0.00
Lasht	22.10±1.02^c^	19.66±2.32^a^	8.33±0.22^d^	0.07±0.00^a^	2.47±0.09^a^	33.38±0.37^d^	5.30±0.10^c^	7.67±10.39^a^
Shahani	31.61±0.81^a^	16.17±0.22^b^	10.89±0.11^b^	0.000	1.48±0.13^a^	34.40±0.82^c^	5.42±0.07c	0.00±0.00
Maktub	24.71±0.35^b^	13.29±0.27^c^	10.94±0.54^b^	0.000	2.26±0.04^a^	41.97±0.72^b^	6.33±0.07^b^	0.46±0.07^a^
Khasuee	25.72±2.30^b^	12.42±0.72^c^	9.53±0.85^c^	0.000	2.31±0.29^a^	44.68±1.62^b^	5.31±0.27^c^	0.00±0.00

Values in the same column bearing different superscripts are significantly (p ≤ 0.05

Table 3 shows the average tocopherol content in date seed oil cultivars based on one-gram oil seed. Shekar had the least tocopherol content in one-gram oil (10.09 μg vit E/g oil) and the maximum amount observed in Shahabi 2 (33.86 μg vit E/g oil). In this study, a positive correlation also observed between tocopherol content in oil and seed cultivars.

**Table 3:** The average content of tocopherol in 14 different of Iranian date seeds oil

**Cultivar**	**Mean (μg vit E/g oil)**	**Mean(μg vit E/g kernel)**
Shahabi 2	1.40 ± 33.86	18.81 ± 0.08
Shahabi	0.06 ± 17.22	0.82 ± 0.00
Goftar	0.44 ± 14.54	0.91 ± 0.02
Zahedi	0.39 ± 12.66	20.79 ± 0.0
Kabkab	0.15 ± 17.32	0.79 ± 0.00
Shekar	0.47 ± 10.09	0.61 ± 0.02
Kabkab Dalki 2	0.08 ± 11.66	0.70 ± 0.00
Majul	0.57 ± 16.70	1.18 ± 0.01
Khenizi	0.18 ± 12.25	0.72 ± 0.01
Sayer	1.36 ± 25.48	1.81 ± 0.10
Lasht	0.44 ± 27.86	1.87 ± 0.03
Shahani	0.51 ± 17.45	1.12 ± 0.03
Maktub	17.42 ± 0.03	1.34 ± 0.00
Khasuee	22.04 ± 0.79	1.00 ± 0.03

In view of the widespread cultivation of dates in Iran, this study was conducted to evaluate some quality indices, fatty acids, mean tocopherols and oxidative stability of fourteen date seed oils. These cultivars are well known owing to their common preference, popularity, economic price, as well as the high availability throughout the year.

In general, date seed oil is characterized by the presence of five major fatty acids (oleic, lauric, myristic, linoleic and palmitic) which together composed about 95% of the total fatty acids. Besbes *et al. *in 2004 (3) studied the chemical composition of two date seeds and reported oleic acid as the major fatty acid by 41.3% and 47.7% content. Lasht and shekar cultivars in the present study by 33.38 ± 0.37% and 51.40 ± 1.13% had the least and the highest oleic acid content respectively. Al-Hooti *et al. *in 1998 (15) found a higher oleic acid amount (53.3-58.8%) in United Arab Emirate cultivars. In their work, the date seed cultivars can be classified to oleic-lauric or oleic-linoleic oil based on two major fatty acids. The Iranian cultivars in this paper were oleic-lauric oils and the linoleic acid was the minimum among five major fatty acids. The third prevalent fatty acid in the Al-Hooti and Besbes studies was linoleic, but myristic acid in our work reported as a third major fatty acid. The highest linoleic acid contents in this work are reported in Goftar, Kabkab, Kabkab Dalki2 and Majul cultivars as about 7.3% and it is not comparable with those reported by Besbes for Allig cultivar (21.0 ± 0.29%).

This study showed that Shekar cultivar was rich in USFA; so this is a direct implication use of this cultivar since the USFAs decline serum cholesterol levels and subsequently protect human against cardiovascular disease (17). Moreover, the quality of oil and its use depends on the proportion of oleic and linoleic acid (18).

Shahani cultivar had the maximum percentage of SFAs due to its high level of lauric acid. Hence, this cultivar is suggested for industrial usages.

The oxidative stability of date seed oil was higher than common vegetable oils due to the low content of linoleic and lack of linolenic as a PUFA acid in order to Cox value formula (Table 1).

As mentioned previously, Cox value column shows that Lasht cultivar could easily be conserved since its oxidative stability is more than the other ones. The mean content of studied date seeds Cox value (1.08) is lower than sunflower (6.48), sesame (6.27), rice bran (4.37) and pistacia atlantica (3.99, 4.13) (19, 20). These values revealed that date seed oil is more resistant to oxidation compared with mentioned oils.

Oxidation procedure not only reduces the food quality and nutritional value but also increases the cancer and cardiovascular diseases and in this paper, the Lasht cultivar had the minimum desire to the oxidation (Table 1). It is clear that most of the studied cultivars consist of Shahabi 2, Goftar, Zahedi, Shekar, Majul, Khenizi, Sayer and Khasuee, contain more unsaturated than saturated fatty acids, but some minor variation were found in the fatty acid profile from one cultivar to another and these differences could be due to the genetic variation. Shahabi2 is the richest one among the other cultivars according to the tocopherol content. The average tocopherol of date seeds was 18.32 ± 6.78 μg vit E/g oil and is comparable with *α*-tocopherol content of apricot seeds in Yildirim research, which was from 3.10 μg/g to 22.45 μg/g (21). In addition, the date seeds tocopherol amount of present study is less than grape seeds amount. Wie *et al*. in 2009 studied the vitamin E content of 14 different grape seed cultivars. The total concentration of grape seed tocopherol and tocotrienol was in the range of 353-688 μg/ g oil (22).

## Conclusion

Cultivars used in this study are commercially important in production. The results of the present investigation show that the Iranian dates are important regarding their fatty acid composition and remarkable high amount of tocopherol. The results can provide an extra income and may contribute to have good nutritional values of this product.
